# Suspected melanoma only when the lesion is greater than 6mm may harm patients

**DOI:** 10.1590/S1679-45082015AO3436

**Published:** 2015

**Authors:** Renato Santos de Oliveira, Daniel Arcuschin de Oliveira, Murilo Costa Souza, Mariane da Silva, Mireille Darc Cavalcanti Brandão

**Affiliations:** 1 Hospital Israelita Albert Einstein, São Paulo, SP, Brazil.; 2 Faculdade de Medicina Santa Marcelina, São Paulo, SP, Brazil.; 3 Universidade Nove de Julho, São Paulo, SP, Brazil.; 4 Clínica Prof. Dr. Renato Santos, São Paulo, SP, Brazil.

**Keywords:** Melanoma/diagnosis, Melanoma/prevention & control

## Abstract

**Objective:**

To analyze the distribution of larger diameter in the pathological report of cutaneous melanoma patients.

**Methods:**

Data were obtained from patients seen from 1994 to 2015. Date, sex, age, maximum diameter, histological subtype, primary site, microscopic thickness, mitoses, ulceration, vertical growth phase, and regression were the variables studied. This study was approved by the National Ethics Committee - Brazil Platform. Patients were grouped into smaller diameter (≤6mm) and larger diameter (>6mm). The statistical analysis used the χ^2^ test (p<0.05).

**Results:**

Of the 292 patients analyzed, 123 were seen between 1994 and 2004, and 169 between 2005 and 2015; in that, 151 women and 141 men, mean age of 52 years. The diameters ranged from 2 to 76mm (mean of 14mm), 81 patients with smaller diameter (≤6mm) and 211 with larger diameter (>6mm). Out of 81 patients with smaller diameter, 29 had invasive melanoma, while 179 of the 211 with larger diameter were invasive. A difference was observed in frequency of vertical growth phase.

**Conclusion:**

Pigmented skin lesions with diameter smaller than 6mm should not be an excluding factor for biopsies, especially when patients present risk of developing skin cancer.

## INTRODUCTION

Cutaneous melanoma (CM) has a high mortality rate and its incidence has increased worldwide in recent decades. In Brazil, the National Cancer Institute *José de Alencar Gomes da Silva* (INCA) estimated that 5,890 new cases would occur in 2015.^(1)^In most cases, the patient complains of a new spot on the skin or a spot (a preexisting melanocytic nevus) that is changing in size, shape, or color.

The so called “ABCD” criteria were first proposed in 1985, with the following features: A for Asymmetry; B for Border (the edges are irregular or blurred); C for Color (mixed patterns); D for Diameter (>6mm). When these criteria are present in melanocytic lesions, melanoma is suspected and biopsies are indicated for its detection in early stages.^(2,3)^

Several studies warned about the large number of patients with melanoma tumor smaller than 6mm across its larger diameter.^(4,5)^ Over the past two decades, increased public awareness and the advancement of diagnostic techniques in clinical practice resulted in the diagnosis of smaller and earlier melanomas. Due to its high ability to metastasize and the consequent high mortality rate, and since it is highly curable with simple surgeries in its early stages, it is crucial not to delay the diagnosis of CM.

## OBJECTIVE

To analyze the distribution of the larger diameter of melanomas in pathology reports of cutaneous melanoma patients.

## METHODS

Data were obtained from pathological reports of CM patients seen at the *Clínica Prof. Dr. Renato Santos*, in the city of São Paulo (SP), which is specialized in skin cancer, from February 1994 to March 2015. This clinic attended insured or private adult patients, mainly from the metropolitan area of São Paulo. According to the consolidated opinion of the Research Ethics Committee (CEP) Platform Brazil, the Informed Consent Term was dismissed, because this was a retrospective data collection from medical records. We excluded the cases in which it was not possible to obtain the measurement of the larger diameter of the primary melanoma.

The variables of interest that served as the basis for the statistical analysis were date of the pathological report (two periods: 1994-2004 and 2005-2015), sex, age (in the report), maximum diameter of the lesion, pathological subtype, primary site (head and neck, upper limbs, lower limbs, and trunk), microscopic thickness (Breslow), mitotic index, ulceration, growth and regression phase. This study was initiated after approval by the National Research Ethics Commission (CONEP) in Brazil Platform, resolution number 1082206, CAAE: 44704715.6.0000.5477.

The patients were separated according to the values of the largest diameter of the primary lesion into a smaller diameter group (≤6mm) and a greater diameter group (>6mm). The data were entered into a spreadsheet for statistical analysis (Excel, Microsoft Corp., Redmond, WA, USA), in 2x2 crosstabs containing the variables of interest, with their calculated frequencies and the application of the χ^2^ test, selected due of the sample size (p<0.05).

## RESULTS

Of the 382 CM patients seen, it was not possible to obtain the measurement of the primary CM diameter in 90 of them, who were excluded. Of the 292 patients analyzed, 123 were seen from 1994 to 2004, and 169 between 2005 and 2015. In the first period, 28 patients had smaller diameter CM (28/123; 23%), whereas in the second period, 53 of 169 patients had “smaller” diameter melanomas (31%). This difference in incidence was statistically significant.

Of the 292 patients analyzed, 151 were female and 141 were male, and there were 44 cases of smaller diameter (29%) among female patients and 37 cases of greater diameter (26%) among male patients.

The age range was 18-91 years (mean, 52 years; median, 53 years). Among patients with smaller diameter, the age range was 22-88 years, and among those with greater diameter, it was 18-91 years.

The most frequent site of the primary melanoma was the limbs, both among patients with smaller diameter (35 of 81 patients; 43%) and with greater diameter (82 of 211 patients; 39%). The superficial spreading melanoma was the most frequent histological subtype, both in the group of smaller diameter (38 of 81 patients, 47%) and in the group of greater diameter (70 of 211 patients, 33%).

The diameters of the melanomas ranged from 2 to 76mm, with a mean of 14mm and a median of 10mm; 81 patients (28%) had diameter ≤6mm, and 211 (78%) had diameter >6mm.

Of the 81 patients with smaller diameter, 29 (36%) had invasive melanoma, whereas among 211 patients with greater diameter, 179 were invasive (85%). The statistical analysis of the frequency of invasion in melanomas with smaller diameter compared to the frequency of invasion in melanomas with larger diameter was significant (p<0.05).

One or more mitoses per square millimeter occurred in 7 of the 81 (9%) patients with smaller diameter, and in 49 of the 211 patients with greater diameter (23%). Ulceration occurred in 4 of 81 (5%) patients with smaller diameter, and in 22 of 211 (10%) patients with greater diameter.

Among the 81 patients with smaller diameter, 33 (41%) had melanomas in the vertical growth phase, whereas in the 211 patients with diameter >6mm, 129 (61%) had melanoma in the vertical growth phase. The statistical analysis comparing the frequency of vertical growth phase in the two diameter groups was significant (p≤0.05).

## DISCUSSION

The cutaneous melanomas treated in a specialized clinic do not represent the epidemiology of those diagnosed in the general population, but this does not invalidate the fact that a significant number of patients (28%; 81 of the 292 patients studied) were diagnosed with melanomas with diameters ≤6mm.^(6,7)^

Although a specimen shrinkage occurs after the excisional biopsy and the fixation for pathological examination, the measurement of the primary CM diameter has a direct correspondence with the primary tumor *in vivo*. The shrinkage of a surgical melanoma specimen is 15-25% depending on the age of the patient.^(8)^ Supposing that a maximum retraction value (25%) occurred in our study, we still would have in our series 42 cases of melanoma with a diameter ≤5mm corresponding to a diameter <6mm *in vivo*.

In the second period (2005-2015), there was a higher percentage of melanomas with diameter >6mm than in the first period. The percentage of melanomas with diameter ≤6mm from 1994 to 2004 (23%) was significantly lower than that from 2005 to 2015 (31%). The greater understanding of preventive attitudes in the general population and among physicians, as well as the advances in diagnostic methods (dermoscopy, videodermoscopy and body mapping), must have contributed to this. There was no significant different distribution between the two groups of patients with greater and smaller diameters in relation to sex and age, and these two variables showed values close to those found in the world population (higher incidence over 50 years and ratio nearly 1:1 between sexes).

The most frequent site of the primary melanoma was the limbs, and the superficial spreading melanoma was the most frequent histological subtype both among patients with smaller and greater diameters.

Of the 81 patients with smaller diameter, 29 (36%) had invasive melanoma, whereas among 211 patients with greater diameter, 179 were invasive (85%). The statistical analysis of the frequency of invasion in melanomas with smaller diameter compared to larger diameter was significant (p≤0.05). This corroborates the efforts to encourage an early diagnosis because the treatment of a less invasive melanoma provides higher cure rates and requires smaller surgeries.

One or more mitoses per square millimeter occurred in 7 of the 81 (9%) patients with smaller diameter, and in 49 of the 211 patients with greater diameter (23%). This difference was significant. A mitotic rate ≥1/mm^2^ denotes a melanoma with high risk of metastasis, particularly for thin melanomas (Breslow<1.0mm). Ulceration occurred in 4 of 81 (5%) patients with smaller diameter, and in 22 of 211 (10%) patients with greater diameter.

Among the 81 patients with smaller diameter, 33 (41%) had melanomas in the vertical growth phase, whereas in the 211 patients with greater diameter, 129 (62%) had melanoma in the vertical growth phase. The statistical analysis comparing the frequency of vertical growth phase in the two diameter groups was significant (p≤0.05). Patients with diameter >6mm had higher frequency of melanoma in the vertical growth phase, indicating a later stage in the natural history of the disease.^(9)^

The patients themselves or family members detected most cases of CM.^(10-12)^ Our results point to an important limitation of the ABCD rule, as a prevention public warning, and reinforce the studies of other authors, indicating the need to reconsider the use of the diameter criterion (>6mm). It should be taken into consideration the number of cases of CM with diameter <6mm.

## CONCLUSION

A diameter <6mm in pigmented lesions should not be an excluding factor in cases in which melanoma is suspected. The current ABCD rule is fallible regarding the variable “diameter”.
