# Supplementary material for: Suspected melanoma only when the lesion is greater than 6mm may harm patients
Source: Einstein (Sao Paulo). 2015 Oct-Dec;13(4):506–9. doi: 10.1590/S1679-45082015AO3436 (PMC4878622; doi:10.1590/S1679-45082015AO3436)
Supplement: Translation

# Translation: Considerar diâmetro apenas maior que 6mm como suspeita de malignidade de melanoma pode prejudicar pacientes

**Authors:** Renato Santos de Oliveira, Daniel Arcuschin de Oliveira, Murilo Costa Souza, Mariane da Silva, Mireille Darc Cavalvanti Brandão

**Affiliations:** 1 Hospital Israelita Albert Einstein, São Paulo, SP, Brasil.; 2 Faculdade de Medicina Santa Marcelina, São Paulo, SP, Brasil.; 3 Universidade Nove de Julho, São Paulo, SP, Brasil.; 4 Clínica Prof. Dr. Renato Santos, São Paulo, SP, Brasil.

**Keywords:** Melanoma/diagnóstico, Melanoma/prevenção & controle

## Abstract

**Objetivo:**

Analisar a distribuição do maior diâmetro reportado no laudo histopatológico de portadores de melanoma cutâneo.

**Métodos:**

Os dados foram obtidos de pacientes atendidos de 1994 a 2015. Data, sexo, idade, diâmetro máximo, subtipo histopatológico, sítio primário, espessura microscópica, mitoses, ulceração, fase de crescimento e regressão foram as variáveis estudadas. O estudo foi aprovado pela Comissão Nacional de Ética em Pesquisa na Plataforma Brasil. Os pacientes foram agrupados em diâmetro menor (≤6mm) e maior (>6mm). Análise estatística utilizou o teste χ^2^ (p<0,05).

**Resultados:**

Dos 292 pacientes analisados, 123 foram atendidos entre 1994 e 2004, e 169 entre 2005 e 2015, sendo 151 mulheres e 141 homens, com média de idade de 52 anos. Os diâmetros variaram de 2 a 76mm (média de 14mm), sendo 81 pacientes com diâmetro menor que 6mm e 211 com diâmetro maior. Dos 81 pacientes com diâmetro menor, 29 apresentavam melanoma invasivo, enquanto 179 dos 211 com diâmetro maior eram invasivos. Houve também diferença de frequência da fase de crescimento vertical.

**Conclusão:**

Diâmetro de lesões pigmentadas menor que 6mm não deve ser fator excludente para realização de biópsias, especialmente para paciente de risco para câncer de pele.

## INTRODUÇÃO

O melanoma cutâneo (MC) apresenta alta mortalidade, e sua incidência tem aumentado mundialmente nas últimas décadas. No Brasil, Instituto Nacional do Câncer José de Alencar Gomes da Silva (INCA) estimou a ocorrência de 5.890 casos novos para 2015.^(1)^ Na maioria dos casos, o paciente se queixa do surgimento de uma nova lesão pigmentada na pele ou de modificações de tamanho, forma ou cor de um nevo melanocítico preexistente.

Assimetria (A), bordas irregulares ou mal definidas (B), coloração mista (C), diâmetro >6mm (D) são os chamados critérios “ABCD”, propostos em 1985. Quando presentes em lesões melanocíticas, levam à suspeita de melanoma, indicando biópsias das mesmas e sua detecção em fases iniciais.^(2,3)^

Muitos trabalhos já alertaram para o expressivo número de pacientes portadores de melanoma com tumores cujo maior diâmetro é menor do que 6mm.^(4,5)^Durante as últimas duas décadas, o aumento da consciência pública e o avanço de técnicas diagnósticas na prática médica resultaram no diagnóstico de melanomas menores e mais precoces. Devido à sua alta capacidade de produzir metástases e a consequente mortalidade, e sendo altamente curável com cirurgias simples em sua fase inicial, é crucial que não se retarde o diagnóstico do MC.

## OBJETIVO

Analisar a distribuição do maior diâmetro reportado no laudo histopatológico de portadores de melanoma cutâneo.

## MÉTODOS

Os dados foram obtidos a partir de laudos histopatológicos de pacientes portadores de MC atendidos na Clínica Prof. Dr. Renato Santos, localizada em São Paulo (SP), especializada em oncologia cutânea, de fevereiro de 1994 a março de 2015. Essa clínica atendia pacientes adultos, conveniados ou particulares, provindos principalmente da grande São Paulo. De acordo com o Parecer Consubstanciado do Comitê de Ética em Pesquisa (CEP) Plataforma Brasil, houve dispensa do Termo de Consentimento Livre e Esclarecido, por se tratar de coleta retrospectiva de dados em prontuários. Foram excluídos os casos em que não foi possível obter o valor da medida do maior diâmetro do melanoma primário.

As variáveis de interesse que serviram de base para análise estatística foram data do laudo histopatológico (dois períodos: de 1994 a 2004, e de 2005 a 2015), sexo, idade (na ocasião do laudo), diâmetro máximo da lesão, subtipo histopatológico, sítio primário (cabeça e pescoço, membros superiores, membros inferiores e tronco), espessura microscópica (Breslow), índice mitótico, ulceração, fase de crescimento e regressão. Este estudo foi iniciado após aprovação pela Comissão Nacional de Ética em Pesquisa (CONEP) na Plataforma Brasil, com parecer número 1.082.206, CAAE: 44704715.6.0000.5477.

Os pacientes foram agrupados de acordo com os valores do maior diâmetro da lesão primária em menor (≤6mm) e maior (>6mm) diâmetro. Os dados foram inseridos em uma planilha para análise estatística (*Excel*, *Microsoft Corp*., Redmond, WA, Estados Unidos), tendo sido criadas tabelas cruzadas 2x2, contendo as variáveis de interesse, com o cálculo de suas frequências e aplicação do teste χ2, escolhido em função do tamanho das amostras (p<0,05).

## RESULTADOS

Dos 382 pacientes portadores de MC atendidos, não foi possível obter o valor da medida do diâmetro do MC primário de 90, que foram excluídos. Dos 292 pacientes analisados, 123 foram atendidos de 1994 a 2004, e 169 entre 2005 e 2015. No primeiro período, 28 pacientes apresentavam MC com diâmetro menor (28/123; 23%), enquanto no segundo período, 53 dos 169 pacientes apresentaram MC com diâmetro “menor” (31%). Essa diferença de incidência teve significância estatística.

Dos 292 pacientes analisados, 151 eram do sexo feminino, e 141 eram do sexo masculino, sendo que ocorreram 44 casos de diâmetro menor (29%) entre as pacientes do sexo feminino e 37 casos de diâmetro maior (26%) entre os pacientes do sexo masculino.

A idade dos pacientes variou de 18 a 91 anos (média de 52 anos e mediana de 53 anos), sendo que, entre os pacientes com diâmetro menor, a idade variou de 22 a 88 anos e, entre aqueles com diâmetro maior, a idade variou de 18 a 91 anos.

O sítio mais frequente do melanoma primário foi os membros, tanto entre os pacientes com diâmetro menor (35 dos 81 pacientes; 43%) como entre aqueles com diâmetro maior (82 dos 211 pacientes; 39%). O subtipo histológico disseminativo superficial foi o mais frequente, tanto no grupo de diâmetro menor (38 dos 81 pacientes; 47%) como no grupo de diâmetro maior (70 dos 211 pacientes; 33%).

Os diâmetros dos melanomas variaram de 2 a 76mm, com média de14mm e mediana de 10mm; 81 pacientes (28%) apresentaram diâmetro ≤6mm e 211 (78%) tiveram diâmetro >6mm.

Dos 81 pacientes com diâmetro menor, 29 (36%) apresentavam melanoma invasivo, enquanto que, entre os 211 pacientes com diâmetro maior, 179 eram invasivos (85%). A análise estatística comparando a frequência de invasão nos melanomas com diâmetro menor aos maiores foi significativa (p<0,05).

Uma ou mais mitoses por milímetro quadrado ocorreram em 7 dos 81 (9%) pacientes com diâmetro menor e em 49 dos 211 pacientes com diâmetro maior (23%). Já a presença de ulceração ocorreu em 4 dos 81 (5%) pacientes com diâmetro menor e em 22 dos 211 (10%) pacientes com diâmetro maior.

Dentre os 81 pacientes com diâmetro menor, 33 (41%) tinham melanoma em fase de crescimento vertical, enquanto que, nos 211 pacientes com diâmetro >6mm, 129 (61%) apresentavam melanoma em fase de crescimento vertical. A análise estatística comparando a frequência da presença de fase de crescimento vertical nos dois diâmetros foi significativa (p≤0,05).

## DISCUSSÃO

Os melanomas cutâneos tratados em uma clínica especializada não representam a epidemiologia daqueles diagnosticados na população geral, mas isso não invalida o fato real do significativo número de pacientes (28%; 81 dos 292 pacientes estudados) diagnosticados com diâmetro ≤6mm.^(6,7)^

Embora ocorra um encolhimento do espécime após a biópsia excisional e a fixação para exame histopatológico, a medida do diâmetro do MC primário tem uma correspondência direta com o da lesão primária *in vivo*. O encolhimento de um espécime cirúrgico de melanoma é de 15 a 25%, dependendo da idade do paciente.^(8)^ Considerando a ocorrência em nosso estudo do valor máximo de retração (25%), ainda temos, em nossa casuística, 42 casos com diâmetro ≤5mm, correspondentes a diâmetro <6mm *in vivo*.

No segundo período (de 2005 a 2015), houve uma porcentagem maior de diâmetro >6mm do que no primeiro período. A porcentagem de melanoma com diâmetro ≤6mm no período de 1994 a 2004 (23%) foi significativamente menor do que a que ocorreu entre 2005 a 2015 (31%). O maior conhecimento de atitudes preventivas na população geral e entre os médicos, bem como os avanços dos métodos diagnósticos (dermatoscopia, videodermatoscopia e mapeamento corporal), deve ter contribuído para isso. Não houve distribuição diferente significativa entre os dois grupos de pacientes com diâmetros maior e menor em relação ao sexo e à idade, sendo que estas duas variáveis apresentaram valores próximos aos encontrados na população mundial (maior incidência acima dos 50 anos e proporção quase 1:1 entre os sexos).

O sítio mais frequente do melanoma primário foi os membros e o subtipo histológico disseminativo superficial foi o mais frequente tanto entre os pacientes com diâmetro menor como entre aqueles com diâmetro maior.

Dos 81 pacientes com diâmetro menor, 29 (36%) apresentavam melanoma invasivo, enquanto que, entre os 211 pacientes com diâmetro maior, 179 eram invasivos (85%). A análise estatística comparando a frequência de invasão nos melanomas com diâmetro menor ao diâmetro maior foi significativa (p≤0,05). Isso corrobora a conduta de incentivar o diagnóstico precoce, pois o tratamento do melanoma menos invasivo oferece maior taxa de cura e requerer cirurgias de menor porte.

Uma ou mais mitoses por milímetro quadrado ocorreram em 7 dos 81 (9%) pacientes com diâmetro menor e em 49 dos 211 pacientes com diâmetro maior (23%). Esta diferença foi significativa. Taxa de mitose ≥1/mm2 denota melanoma com alto risco de metástase, particularmente para os melanomas finos (Breslow <1,0mm). Já a presença de ulceração ocorreu em 4 dos 81 (5%) pacientes com diâmetro menor e em 22 dos 211 (10%) pacientes com diâmetro maior, sem diferença estatística significante.

Dentre os 81 pacientes com diâmetro menor, 33 (41%) tinham melanoma em fase de crescimento vertical, enquanto que, nos 211 pacientes com diâmetro maior, 129 (62%) apresentavam melanoma em fase de crescimento vertical. A análise estatística comparando a frequência da presença de fase de crescimento vertical nos dois grupos de diâmetros foi significativa (p<0,05). Os pacientes com diâmetro >6mm apresentaram maior frequência de melanomas em fase de crescimento vertical, evidenciando uma fase mais adiantada na história natural da doença.^(9)^

Os próprios pacientes ou familiares detectam a maioria dos casos de MC.^(10-12)^Nossos resultados apontam para uma limitação importante da regra do ABCD, como mensagem pública de prevenção, e reforçam o trabalho de outros autores para repensar a divulgação do critério diâmetro (>6mm). Deve ser levado em consideração o número de casos de MC com diâmetro <6mm.

## CONCLUSÃO

Diâmetro <6mm de lesões pigmentadas não deve ser fator excludente para suspeitar de melanoma. A regra atual do ABCD é falha na variável “diâmetro”.
